# Using Smart Virtual-Sensor Nodes to Improve the Robustness of Indoor Localization Systems

**DOI:** 10.3390/s21113912

**Published:** 2021-06-06

**Authors:** Guilherme Pedrollo, Andréa Aparecida Konzen, Wagner Ourique de Morais, Edison Pignaton de Freitas

**Affiliations:** 1 School of Engineering, Federal University of Rio Grande do Sul, Porto Alegre 90040-060, Brazil; guilherme.pedrollo@ufrgs.br (G.P.); andreakon@ufrgs.br (A.A.K.); 2 School of Information Technology, Halmstad University, 301 18 Halmstad, Sweden; Wagner.deMorais@hh.se

**Keywords:** neural networks, machine learning, indoor localization, wireless sensor network, virtual sensor

## Abstract

Young, older, frail, and disabled individuals can require some form of monitoring or assistance, mainly when critical situations occur, such as falling and wandering. Healthcare facilities are increasingly interested in e-health systems that can detect and respond to emergencies on time. Indoor localization is an essential function in such e-health systems, and it typically relies on wireless sensor networks (WSN) composed of fixed and mobile nodes. Nodes in the network can become permanently or momentarily unavailable due to, for example, power failures, being out of range, and wrong placement. Consequently, unavailable sensors not providing data can compromise the system’s overall function. One approach to overcome the problem is to employ virtual sensors as replacements for unavailable sensors and generate synthetic but still realistic data. This paper investigated the viability of modelling and artificially reproducing the path of a monitored target tracked by a WSN with unavailable sensors. Particularly, the case with just a single sensor was explored. Based on the coordinates of the last measured positions by the unavailable node, a neural network was trained with 4 min of not very linear data to reproduce the behavior of a sensor that become unavailable for about 2 min. Such an approach provided reasonably successful results, especially for areas close to the room’s entrances and exits, which are critical for the security monitoring of patients in healthcare facilities.

## 1. Introduction

Infants, older adults, and people with mental or physical disabilities might need special attention in healthcare facilities. Consequently, healthcare staff need to know the indoor location of those patients in order to provide adequate care or assistance [1]. One approach for locating people indoors is to use a wireless sensor network (WSN) that can include fixed and mobile nodes to acquire data to compute the target’s location [2,3,4].

Several technologies and methods for indoor positioning systems (IPS) exist in the literature, as recently examined in [5]. Among these technologies, research has explored radio-frequency identification devices (RFIDs), WiFi, Bluetooth, ZigBee, ultrawide band, and sound for IPS. Range, power consumption, cost, latency, and accuracy are the main criteria to compare and select these technologies. Depending on the selected technologies, signal metrics, including received-signal strength indicator (RSSI), channel-state information (CSI), fingerprinting analysis, angle of arrival (AoA), time of flight (ToF), time difference of arrival (TDoA), return time of flight (RToF), and phase of arrival (PoA) are used to estimate the target’s location. RSSI is a widely used technique due to its low cost and no need for additional hardware [6].

Researchers in [2] evaluated WiFi, ZigBee, and Bluetooth as technologies, and fingerprinting analysis, ToA, and RSSI as localization algorithms to enable IPS in a hospital. Besides suggesting WiFi and fingerprinting analysis as the most suitable approach for IPS in a hospital, an important observation in that study was the impact of a nonfunctioning node on overall system stability. One approach to overcome this problem is using machine-learning techniques to create a model for smart virtual sensors based on past data generated by actual sensor nodes. In run-time, and when a given node becomes unavailable, a smart virtual sensor can provide data in place of the unavailable node. Besides IPS, there are many other applications for smart virtual sensors. For example, the researchers in [7] used an artificial neural network (ANN) and data-mining techniques to develop a virtual sensor that effectively generated cylinder pressure data-predicting emissions in a diesel engine.

An ANN is inspired by natural neuronal function, having its origin in the artificial neuron described in [8]. This artificial neuron was used to compose a network called perceptron [9], with only one layer of binary neurons, and it was the first neural network with commercial applications. As a remarkable evolution, the delta rule [10], which was based on squared-error minimization, allowed for the efficient training of a one-layer network with real domain outputs. However, this network could not solve more complex logical problems and perform nonlinear discrimination or approximate nonlinear functions. Multilayer-perceptron (MLP) networks, capable of approximating nonlinear functions and nonlinear discrimination, became possible with the proposal of the backpropagation algorithm by [11], which can calculate internal layer errors, making it possible to train them with the delta rule. According to the universal approximation theorem [12], these networks can precisely approximate any function with a single inner layer and 2n+1 artificial neurons in this layer; *n* is the number of entries. The theorem by [13], however, more related to practical applicability, stated that a neural network with a single hidden layer could approximate any measurable relationship r:Rn−>Rm, where *m* is the number of outputs. However, the recognized ability of functions approximation can be a drawback because the neural network can approximate even the randomness contained in the data. This phenomenon is called overfitting. To avoid overfitting, the most commonly used method is cross-validation [14], which divides the original samples into three sets: training, validation, and verification. The training set is used to train the neural network by successively submitting all of its data to the training algorithm. Each submission may be called a training cycle or epoch. The algorithm continuously evaluates its performance at each training cycle, in addition to with the validation samples. Performance statistics used internally by the performance evaluation algorithm, as a rule, are the sum of the square of errors. When the algorithm perceives itself as adjusting too well for the training samples but not improving the validating paths, it stops the training to avoid losing its generalization capacity. The neural network is then applied to a set of samples (verification sampling) that did not participate in any previous stages of training or the definition of the neural network complexity to test its generalization capability [14]. Using neural networks in this work is connected to its complex and dynamic structure that allows for modelling a wide range of problems, adjusting to different situations, and recognizing patterns.

Observing the applicability of ANNs, this paper presents a method for estimating a target location with only one sensor and a feed-forward ANN when the actual sensor becomes unavailable for a brief period. This paper also presents an algorithm to detect the target’s position within the range of the sensors when the network is working well, as the system uses the data collected during this period to train the ANN further. The proposed method is independent of the employed WSN protocol, and the developed system can work with only one RSSI sensor with considerable accuracy. This work also presents an evaluation of the sufficient number of neurons that the ANN requires to present acceptable behavior when artificially replacing an actual sensor node in a WSN.

The main contributions of this work are summarized as follows:

- using of machine learning and neural networks to implement virtual sensors to replace temporarily unavailable physical sensors nodes in order to maintain the operation of indoor localization systems;
- assessing of the number of neurons in the ANN that are sufficient to provide satisfactory results in keeping the network computing the target’s position.

The remainder of this paper is organized as follows: Section 2 presents a discussion on related work. Section 3 discusses the indoor-localization problem, including dealing with failure in the nodes and an overview of the proposed solution. Section 4 presents the fingerprint calculation. Section 5 presents the proposed system architecture, while Section 6 presents the experimental setup and results. Lastly, Section 7 concludes the study and presents future work.

## 2. Related Works

In [15], the authors proposed a machine-learning-based WSN system for autistic-activity recognition. The system included a wearable device that tracked autistic patients using a global-positioning-sensor (GPS) module and communicated the location to caregivers. However, GPS consumes too much energy and does not work well for indoor positioning [16].

The work presented in [1] proposed a novel indoor-localization system for healthcare environments based on decentralized RSSI that focused on the placement of the nodes to reduce attenuation caused by hospital walls. This work proved that indoor localization is feasible in hospitals. The authors also suggested simultaneously using more than one RSSI for propagation effects.

The researchers in [4] explored an experimental e-health application with WSN to monitor a person’s care needs in a home environment. As part of the system, the authors developed a simple RSSI-based IPS using ZigBee. The person’s care needs were identified by the system using data fusion from pressure sensors in the bed, a fire sensor in the kitchen, and wearable heart-rate monitors. Multisensor data fusion was also explored in [17] in an indoor navigation system for environments such as hospitals. The proposed method fused map information and data from a light sensor and Bluetooth module in a smartphone to estimate indoor location. Compared to localization systems using inertial sensors, the multisensor fusion approach achieved 90% improvement in localization accuracy.

The work reported in [18] described the implementation and analysis of an indoor-location system that also employed multisensor fusion using magnetic-field sensors (MFS), RSS sensors, and images taken with a mobile phone. A deep-learning module interpreted the captured images and modeled the scene, which would later be fused with MFS and RSSI sensor data to estimate a subject’s location.

The proposal presented in [16] used three different machine-learning methods and radio fingerprints for WSN localization. Among kernel-based machine-learning techniques, the authors selected least-squares support vector machine (SVM), support vector regression (SVR), and vector-output regularized least squares (vo-RLS) with centralized RSSI fingerprinting. The authors claimed that their method surpassed the results achieved by the weighted K-nearest-neighbor algorithm.

Regarding noisy fingerprint classification, a multiple layered perceptron (MLP) dealt well with a noisy dataset by identifying and classifying it in [19]. According to [20], a statistical method can also provide good results to correct errors in RSSI for WSNs.

The study presented in [2] investigated techniques for scene analysis (fingerprinting). The study combined time of arrival (ToA) and RSSI multilateration with three different wireless-communication technologies: WiFi, ZigBee, and Bluetooth Low Energy (BLE). The authors used an indoor-positioning system with a fixed anchor and mobile nodes spread through a hospital environment to test the different setups. The authors evaluated the setup according to accuracy and latency. Image processing with WiFi had the best accuracy, but the worst latency and cost. The authors also mapped the space and evaluated the number of anchor nodes necessary for each method, and the consequences of missing or failed nodes.

One approach to deal with unavailable or problematic nodes is to create virtual sensors modeled to behave and produce data using the historical data of an actual node. For example, virtual sensors were used for predicting diesel-engine emissions using cylinder-pressure data in [7]. This work investigated using many kernel-based machine-learning techniques, such as ridge regression, SVM, and vector-output regularized least squares to improve the RSS technique.

The study presented in [21] evaluated three different artificial neural networks (ANN) using root mean square error (RMSE) for IPS using RSSI. The differences in these methods lay in the type of neural network backpropagation, which can be feed-forward, cascade-forward, or Elman. For a distance range of 100 m, Elman backpropagation presented the least RMSE, which was more accurate.

The work reported in [22] described the development of fingerprinting mapping using an RSSI online K-nearest-neighbor algorithm for indoor WiFi services. In [23], the authors used a feature-adaptive online sequential extreme learning machine (another machine-learning technique) for lifelong indoor WiFi localization that could improve its accuracy even with fewer data. A Lagrangean programming neural network was used in [3] to complement a TOA-based approach for indoor-positioning detection.

Aiming at recognizing indoor human activity using high-dimensional sensors and deep neural networks, a fusion of video-camera and radar sensors by a three-dimensional convolutional neural approach was explored in [24]. In contrast, [25] applied radio sensors to fingerprint-based device-free (DF) WiFi indoor localization that coped with noisy channel-state information.

In [26], the proposed CCPos positioning system (CADE-CNN positioning) used a convolutional noise-elimination autoencoder (CDAE) and convolutional neural network (CNN). The authors explained that in the offline stage, the system applied the K-means algorithm to extract the validation set from the complete and online training set; the RSSI was first demineralized, and the CDAE extracted key resources, so the location estimate was issued by the CNN. Reported experiment results showed good performance.

In the solution proposed in [27], a scheme based on the similarity of measures using machine learning in data analysis was used to implement virtual sensors in a sensor cloud. The physical sensors were classified into several categories using historical data, so the k-means algorithm was explored for each class to group those with high similarity. Lastly, a physical sensor representative of each cluster was selected to create the corresponding virtual sensors. The most interesting results are related to the accuracy of the collected data.

Finding objects in a disordered reverberating environment is extremely challenging. The authors in [28] showed that a non-emitting object’s scattering contribution to a reverberant medium suffices to localize the object and demonstrate this finding in the microwave domain. Then, they further simplified the scheme by replacing the temporal degrees of freedom of the broadband measurement with spatial degrees of freedom obtained from wavefront shaping. According to the study, the demonstrated ability to localize multiple noncooperative objects with a single-frequency scheme has potential usage in smart-home applications.

Studying the same problem of locating objects in complex environments, the work reported in [29] revealed that environmental perturbations reduce both the diversity of the dictionary of solutions based on wave fingerprints and their effective signal-to-noise ratio (SNR). These effects are such that they reduce the amount of information that can be obtained per measurement. However, the authors state that this unfavorable effect can be fully compensated by taking additional measurements. The authors showed experiments in which the localization of noncooperative objects is possible even when the scattering strength of the environmental perturbation significantly exceeds that of the target object.

The authors in [30] showed that the precision of localizing a subwavelength object could be improved by several orders of magnitude by simply enclosing it in its far field with a reverberating chaotic cavity. Their experiment results demonstrated that their approach could locate a subwavelength object inside a chaotic cavity using only single-frequency single-pixel measurements.

The work presented in [31] focused on decimeter-level localization with a single WiFi access point (Chronos). The authors investigated whether a WiFi radio could emulate a wide-band multi-GHz radio for the purpose of localization. The proposed solution could compute sub-nano-second time of flight using commodity WiFi cards. As proposed in the paper, by multiplying the time of flight with the speed of light, a MIMO access point could compute the distance between each of its antennas and the client, hence localizing it.

Ubicarse is an accurate indoor localization with zero start-up cost presented in [32]. This system requires no specialized infrastructure nor fingerprinting. Ubicarse allows for handheld devices to emulate large antenna arrays using a new synthetic aperture radar (SAR). The contribution is the ability to perform SAR on handheld devices twisted by their users along unknown paths, and is not limited to localizing RF devices; it combines RF localization with stereovision algorithms to localize everyday objects with no RF source attached to them.

WiTrack2.0 is a multiperson localization system that operates in multipath-rich indoor environments presented in [33]. It pinpoints users’ locations based on the reflections of wireless signals off their bodies. This approach works even if the user is behind a wall or obstruction. The reported results show that it could simultaneously localize up to five people with a median accuracy of 11.7 cm in each of the *x*/*y* dimensions, providing a coarse tracking of body parts.

The work presented in this paper advances the state of the art by proposing an IPS system that can support the failure of a sensor node by replacing it with a virtual ANN sensor that provides the expected positioning data. This approach (1) overcomes the problems and gaps associated with handling failures, (2) provides a low-cost alternative to the related work, as it demands sophisticated hardware, and (3) does not present problems regarding privacy issues, for example, by not handling images of a scene.

## 3. Problem Statement and Proposed Solution Overview

As discussed in the introduction, certain people need special healthcare attention. In order to provide such services, healthcare personnel must constantly know where these people are, especially for urgent needs. Assuming a WSN to monitor the patient’s location in this environment, it is possible to model it as a Cartesian coordinate plane where sensor nodes are displaced and can provide location information of the tracked people. The literature presents many tactics to discover the location of tracked objects of people in both indoor and outdoor environments. This work used an RSSI-based solution for the indoor scenario. This solution based on WSN explores spatial diversity of the distribution of nodes in a monitored environment. The goal of the system is not to compute accurately the positioning of a person inside a room, but to provide information such as if the monitored person left a room or if the person is walking by a restricted area.

As in any WSN, a sensor may fail in providing expected data due to several reasons, such as battery depletion, interference, or hardware problems. The unavailability of a sensor node impacts the location or tracking system because expected-positioning data are not provided. This work is, therefore, focused on replacing these missing positioning data so that the location service is not disturbed.

In order to address the problem of an unavailable node, this work proposes a neural network approach that can temporarily virtually replace a real sensor when it becomes unavailable through time-series predictions. This is a fault operation mode of the system. The neural network separately estimates the *X* and *Y* Cartesian coordinates of the target in the function of the measured RSSI and the learnt data. Since the two sensors (the virtual and real one) are theoretically equal, this paper presents a comparative analysis of system behavior when both sensors are on and when just the artificial one is activated.

The proposal is based on fingerprint calculation, which requires patients to carry extra equipment. Alternatives using visual analysis or proximity sensors could be used. However, the former has problems regarding the privacy of the monitored person. At the same time, the latter relies on sensors that may much vary in terms of accuracy considering the type of hardware that is used to deploy the system [34].

The proposed solution considers the usage of devices that are can detect a difference in the received RSSI with enough accuracy to support the detection of movement based on the difference of two consecutive RSSI measurements. Moreover, considering the findings reported in [35], this work considers that the combination of the position where sensors are deployed in the monitored environment and the position in which the person carries the carry-on device is such that the possible negative effects due to the antennas’ angular configuration and a person’s body are negligible.

This work assumes that clock synchronization among sensor nodes is solved by using approaches such as [36], which provide the necessary accuracy to enable the proposed solution to work appropriately.

There is a small correlation (0.305) between distance measured by the bother sensors, but it is too small to dismiss the usage of another sensor or a resource, such as the technique proposed here, to replace the failing sensor. This correlation was found by comparing the data registered for each of the measured positions from one sensor with the corresponding measured position of the other sensor through MATLAB function corr(), which establishes the pairwise correlation coefficient between each pair of vectors, which in this work comprises the time series of the detected positions by each sensor.

## 4. Fingerprint Calculation

The target node exchanges messages with the anchor nodes, which detect the RSSI of the target. The nearer the target is to the anchor, the greater the RSSI is. From this, it is possible to calculate the distance (*r*) of the target according to the Friss equation (used to find the optimal received power at an antenna from basic information about the transmission), shown in (1), which relates this distance with the power of the $P_{t}$ transmitter, received power $P_{r}$, directivity $D_{t}$, distance $D_{r}$ between ($P_{t}$) and ($P_{r}$), and signal frequency λ. Fixing all other parameters, it is possible to establish a direct relation between distance and received power.

(1) $$\left(P_{R}/P_{t}\right)=D_{t}*D_{r}*{\left(\frac{\lambda }{\left(4*\pi *r\right)}\right)}^{2}.$$

**Figure 1:**
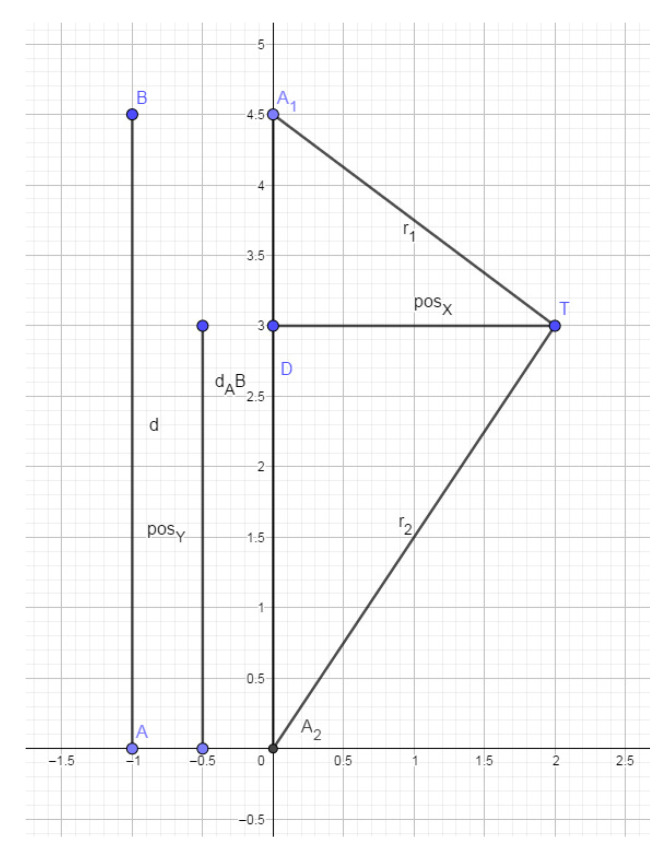
How sensors identify target position.

Following the scenario representation in Figure 1, the distance between a sensor $A_{1}$ and a target *T* is $r_{1}$, while the distance between T and another sensor $A_{2}$ is $r_{2}$. Variable $pos_{X}$ represents the position of *T* regarding the horizontal Cartesian axis (X), while $pos_{Y}$ is its analog to the vertical Cartesian axis (Y). So, in this work, the sensors were considered to both be on the Y-axis. Therefore, the distances between the anchor and mobile nodes form a triangle, represented in Figure 1.

Trigonometric algebra can calculate the $pos_{X}$ and $pos_{Y}$ from the already known distance between nodes. Considering the distance between two sensors (*d*), the greater triangle from Figure 1 is divisible into two rectangle triangles. Using the Pythagorean theorem, two equations are achieved, (2) and (3):

(2) $$r_{2}=\sqrt{\left(pos_{X}^{2}+pos_{Y}^{2}\right)},$$

(3) $$r_{1}=\sqrt{\left(pos_{X}^{2}+{\left(d-pos_{Y}\right)}^{2}\right)}$$

Reorganizing (2) to isolate the $pos_{Y}$ variable, it becomes (4):

(4) $$pos_{Y}=\sqrt{r_{2}^{2}-pos_{X}^{2}},$$

By substituting (4) into (3) and reorganizing it, it becomes Equation (5), which allows for the calculus of $pos_{X}$.

(5) $$pos_{X}=\sqrt{{\left(\frac{r_{2}^{2}-r_{1}^{2}-d^{2}}{\left(-2*d\right)}\right)}^{2}+r_{2}^{2}}.$$

Now, the definition of $pos_{Y}$ is possible through Equation (4).

This calculus yields two possible positions: one was discarded outside the room, and the other was mirrored in the triangle’s central X axis.

## 5. System Architecture

**Figure 2:**
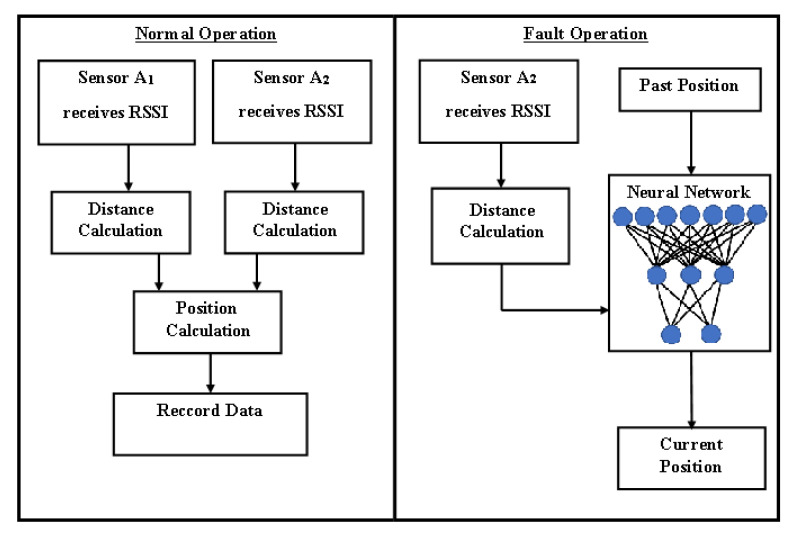
Proposed system architecture.

The system presented in this work has normal and fault-operation modes. First, at normal operation mode, the two anchor node sensors track the mobile sensor (target *T*) attached to the patient and retrieve its RSSI. Next, they calculate its correspondent distance. The system then calculates the target position represented by its Cartesian coordinates, as presented in Section 3. Next, the calculated position is recorded as data for the machine-learning algorithm. Lastly, the cycle restarts 1 second later with another instance of data acquisition. If one of these sensors fails, the neural network is activated. A proximity sensor at the door is also activated in order to know if someone enters the room. So, this neural network receives the starting position, speed, and distance acquired by the sensor $A_{2}$. From these data, the trained machine-learning algorithm estimates the position of the target. Figure 2 presents the system operation under normal and fault conditions.

This neural network functions as a virtual sensor to simulate the information that would be retrieved by a faulty sensor $A_{1}$ during its failure considering its past data and current information from sensor $A_{2}$. The neural network is trained with the same inputs as those that it needs for operating. The neural network comprises an input layer, at least one inner layer (also called a hidden layer), and one output layer.

The input layer receives the data and introduces them to the other neurons. In this study case, the input layer needed 7 neurons to work properly, as there were 7 inputs besides the bias, which was considered to have the value of 1. Thus, this neural network received past positions (x(t−1), y(t−1)), (x(t−2), y(t−2)), current and past distances calculated from sensor $A_{2}\left(r\left(t\right),r\left(t-1\right),r\left(t-2\right)\right)$. Comparing the last two positions allows for the machine-learning algorithm to estimate the movement’s speed and its inertial tendency.

**Figure 3:**
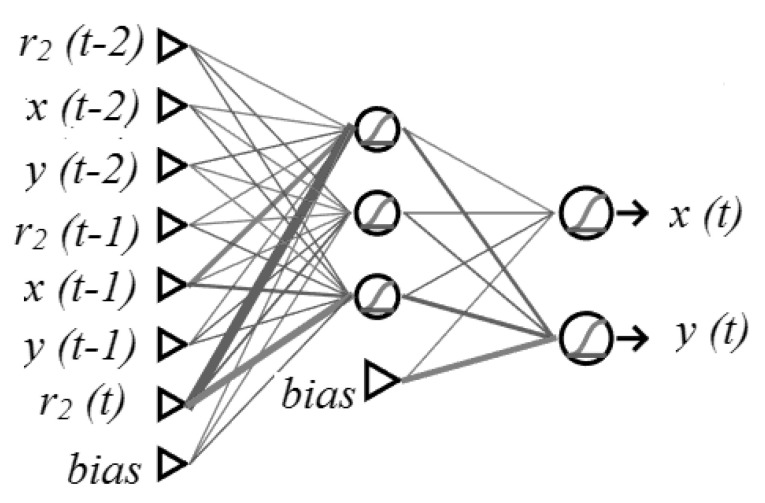
Neural network composed of input, hidden, and output layers.

The output layer consisted of two neurons that provide the estimations of the patient’s position. The outputs of the algorithm are coordinates in the *X* and *Y* axes for each interaction step. Figure 3 shows this neural network.

The ANN calculates the following possible positions based on the known position, trajectory, and distance radius from the active sensor. ANN training enables it to choose the most likely among the next possible positions considering trajectory targets that it had found.

A neural network can have one or more hidden layers. When it has more than one hidden layer, it receives the name of a deep neural network capable of more complex estimation and predictions. However, such a network requires substantial amounts of data that are not always available, as in the present case study. Therefore, the present study used an MLP approach with only one hidden layer. This approach was enough to track the person’s position given by the dataset, as shown in Section 6. Remarkably, additional sensors would improve detection accuracy, as the ANN training data would be more robust.

In order to avoid overfitting problems, it is necessary to use cross-validation. This technique divides a dataset into training (255 samples), validation (121 samples), and verification (105 samples) sets. The first set is used to train the ANN by successively submitting all of its data to the training algorithm. Each submission may be called a training cycle or epoch. The algorithm continuously evaluates its performance at each training cycle and with the validation samples. Performance statistics used internally by the performance-evaluation algorithm, as a rule, are the sum of the square of errors. When the algorithm perceives itself as adjusting too well for the training samples but not improving for the validating paths, it stops the training to avoid losing its generalization characteristics. The neural network is then applied to a set of samples (verification sampling) that did not participate in any previous stages of training or the definition of the neural network’s complexity to test its generalization capability [14].

## 6. Experiments and Results

This section presents neural network behavior as a virtual sensor for indoor human tracking. This study compared the positions tracked by the machine-learning algorithm with the acquired positions using two sensors ($A_{1}$ and $A_{2}$) from the dataset. Experiments were conducted via simulations using the standard resources of MATLAB 2012 to program the neural network that functioned as a virtual sensor based on data from the real sensors, including past data from the compromised sensor.

First, repetitive tests were conducted to determine the number of neurons at the hidden layer of the neural network that provides a better approximation of the behavior of the sensors. The used criteria to choose the ANN complexity, represented by the number of neurons in the inner layer, is the complexity that did not present performance reduction with its application to the validation sampling concerning an oversized network previously trained with cross-validation, which did not show overfitting. Therefore, the resulting network practically presented the same performance (except for the randomness of the indices used for the evaluation) as that of the initial network. For minor complexities, it was verified that the lack of degrees of freedom impairs performance.

### 6.1. Experimental Setup

The scenario in the dataset provided by [37], which is used in this paper, consisting of a standard four-wall room connected by a corridor to another equally sized room. Each wall opposite the corridor entrances had two suspended anchor nodes, one at each corner at 1.5 m of the ground. In [37], the authors established six possible paths and three different room sizes in their acquisitions. This scenario considers that the target had a mobile node in the chest that interacted with the anchor nodes. The system used RSSI to evaluate the distance between mobile and anchor nodes. The corridor was long enough that the sensors of the other room had a reach limited to point *M*. The dataset recorded six different paths.

The present paper considers just one room of the above-described dataset, and one path constrained in this room, which had a length of 12.6 m and a width of 4.5 m. Thus, the simulation employed a simplified open-space environment with fixed sensor positions in this area of interest, i.e., the 12.6 × 4.5 m room, and the effect of the walls, furniture, and other obstacles was not taken in account.

**Figure 4:**
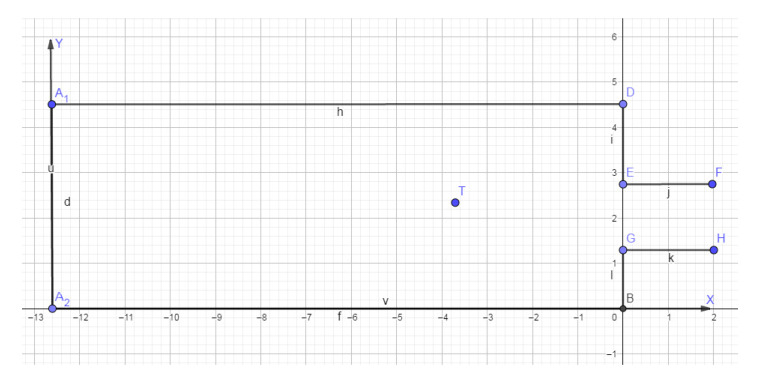
Scenario: room such as the one from the dataset.

The dataset, and thereby this work, consider that a person can move 1 m per second. From the distances of two or more sensors, the system could calculate the mobile node’s position in a Cartesian plane, as follows. Figure 4 presents a scenario used in the present work with anchor nodes $A_{1}$ and $A_{2}$ at its corners. The scope of the current work considers just the second path of the dataset, which did not transcend the limits of this room. All sensors are supposed to be ideal with spherical radiation diagrams, and the effect of the body and any other objects, and any potential interference were not covered in the performed simulations.

**Figure 5:**
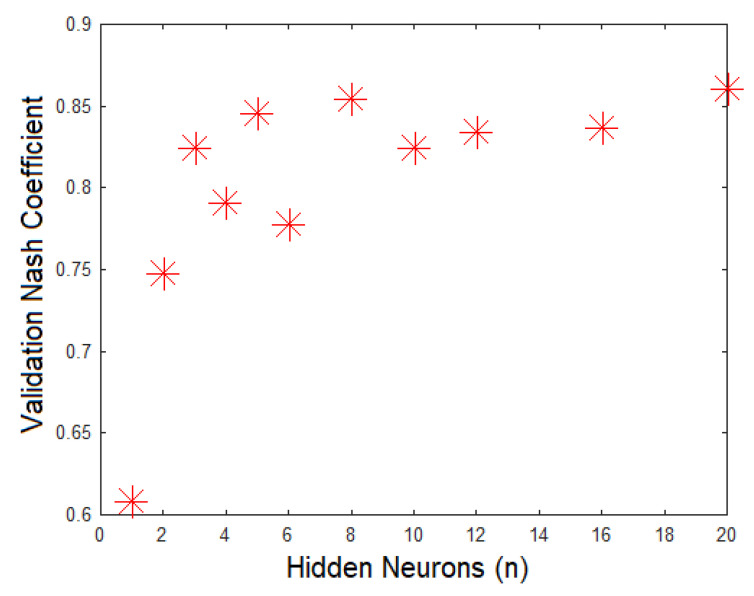
Evaluation of Nash coefficient by number of neurons at the hidden layer.

The statistics used for determining the number of neurons were the Nash efficiency coefficient and the absolute average error between observed and calculated data. The Nash efficiency coefficient explains the proportion of variance in the data that the model can explain. This parameter varies from negative infinite to 1. The better the model is, the closer the Nash coefficient is to 1. Figure 5 presents the Nash coefficient by numbers of neurons at the hidden layer. The Nash coefficient showed a difference when 3 neurons were used instead of 2 neurons. When the neurons in the hidden layer were increased by more than that, the coefficient was not clearly and significantly improved.

**Figure 6:**
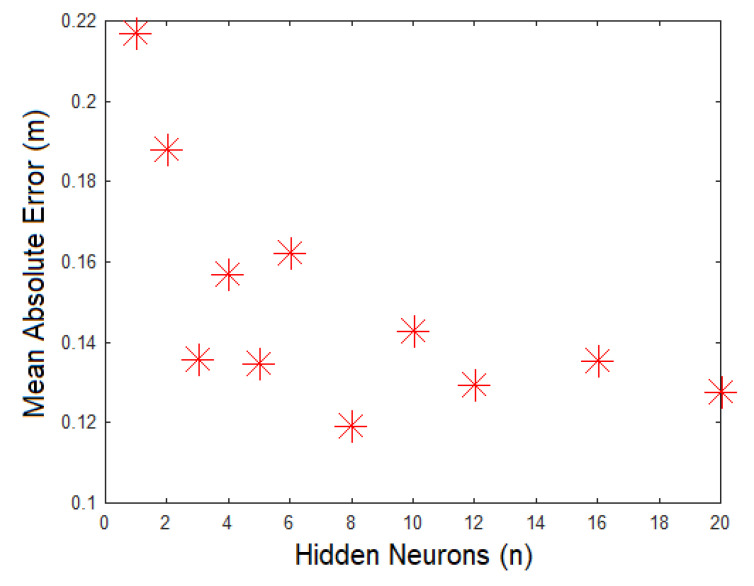
Evaluation of absolute average error by number of neurons at hidden layers.

Figure 6 presents an evaluation by absolute mean error for the same models previously evaluated by the Nash coefficient. The error was more significant when the model had one or two neurons, and started improving with 3 neurons. From that point on, both the average absolute error and Nash coefficient started becoming acceptable. Therefore, the following analysis features results for a model with three neurons at the hidden layer, which had a Nash coefficient value of 0.8246 and an average absolute error of 0.1359, as shown in the graph.

### 6.2. Experiment Results

**Figure 7:**
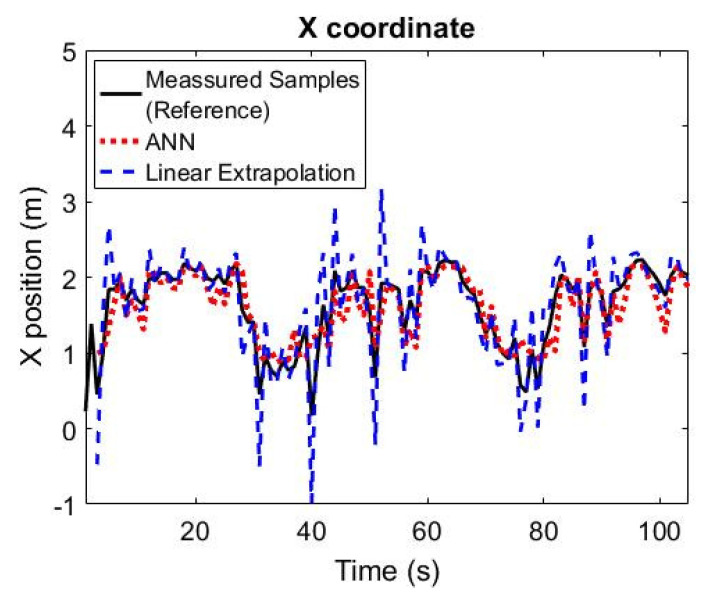
Artificial neural network and linear extrapolation following measured patient path in relation to *X* axis.

Figure 7 shows how the neural network’s time-series prediction algorithm tracks the verification set paths concerning the position of the target on the *x* axis. This figure shows that the neural network followed the target’s position with considerable precision. The model was less accurate for lateral-end positions near the path origin (lower values). On the other hand, as the patient approached the exit (high values), the model more accurately hit their position. For comparison purposes, a simpler model based on linear extrapolation was used as the baseline. The model started by hitting the dashed line that represented the original path. The model’s output starts a bit later than the original signal from the sensors (no line filled following the dashed line in the first moments). This is because the model needed those first sensor data from the original path. The average neural network tracking error in relation to the *X* axis was 0.036 m. Comparing the mean error with the median (0.055 m) revealed that the statistical distribution of errors presented symmetrical behavior, perhaps with a very short tail to the left. The absolute mean error was 0.199 m. This value shows that the neural network calculated the target’s position for the *X* axis with an average error of about 20 cm. The standard deviation (STD) of this error measure was 0.2704 m, while the root-mean-square error (RMSE) was equal to 0.271 m. These statistics show the dispersion that characterizes the statistical distribution of the precision of this artificial-intelligence model. The Nash coefficient was 0.716. For *X* positioning, the simple linear-extrapolation model presented inferior results and superior error, i.e., absolute mean error of 0.263 m, standard deviation error m, RMSE of 0.382 m, and a Nash coefficient of 0.438.

**Figure 8:**
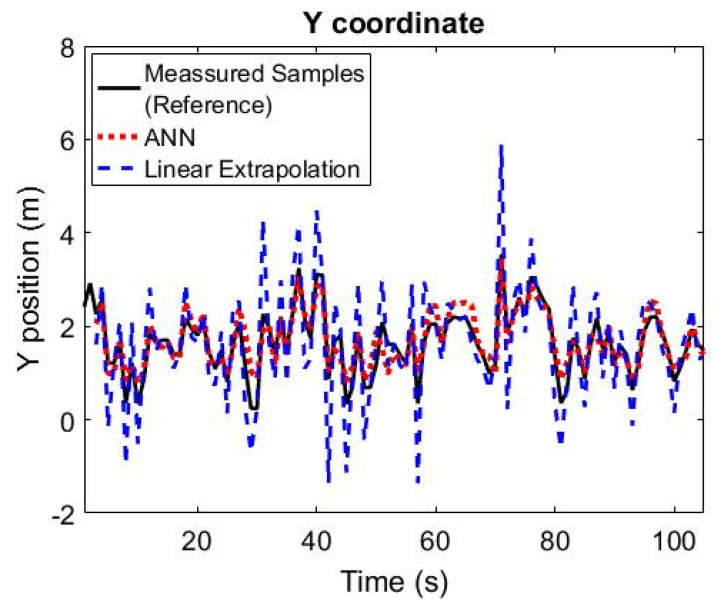
Artificial neural network and linear extrapolation following measured patient path in relation to *Y* axis.

Figure 8 shows the neural network algorithm tracking the verification set paths with respect to the position of the target on the *Y* axis. So, the algorithm tracked the *y*-positioning with even better accuracy than that for the *X* axis. As was noted for the *x* coordinates, the model was less accurate for lateral-end positions near the path origin (lower values), and more accurate as the patient approached the exit (higher values).

**Figure 9:**
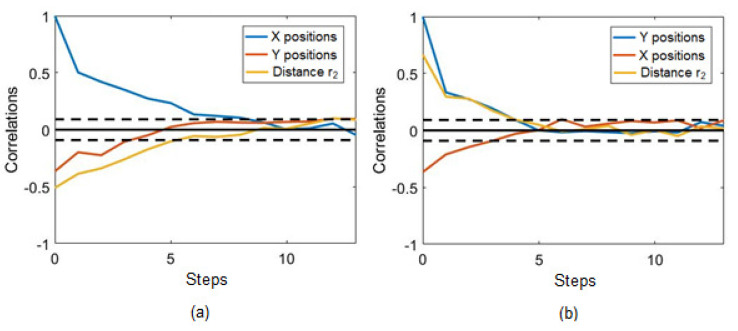
Correlograms of distance r2, and *X* and *Y* positions in relation to (**a**) *X* and (**b**) *Y* positions.

Time-series correlations were performed to evaluate the linear temporal relationships between *X* and *Y* coordinates, and the past time values of these variables and the measured distances of sensor r2. The steps were each of the last discrete positions measured for each variable. The time from one step to another varied according to the individual speed of the target. Thus, decay in the accuracy of the virtual sensor is a function of the steps, and the time of the decay varied with a proportional relation with the steps that the virtual sensor can reproduce and the individual speed of the target. Figure 9a shows the decay of the importance of the past values of *X* and *Y* positions, and distance r2 for the calculation of the current *X* position. The autocorrelation of *X* diminished and became statistically insignificant after 8 steps, though the importance of the two other variables for calculating the *X* position ends before five steps. Figure 9b shows that all variables became statistically insignificant for the calculation of position *Y* after about 5 steps. The significant limits of the correlations, considering a significance level of 0.05, are represented by the dashed lines. These correlograms represent a linear analysis and shown what is expected considering a linear model. However, the ANNs were nonlinear models that may perceive information beyond the linear correlations.

Concerning the *Y* axis, the neural network tracked this position with a mean error of −0.066 m with a median of −0.011 m. Comparing the mean error with the median reveals that the statistical distribution of errors presents symmetrical behavior, maybe with a very short tail to the left, similar to the behavior of the *X* axis. For the ANN, the absolute mean error of the *Y* axis tracking is 0.222 m, its standard deviation is 0.2806 m, the RMSE is 0.2869 m, and the Nash coefficient was 0.820. While the Linear Extrapolation achieved a mean absolute error (MAE) of 0.572, a standard deviation error of 0.752, a RMSE of 0.749 and a Nash coefficient of −0.222. Therefore, linear extrapolation mean absolute error, standard deviation, and RMSE are more than twice the value achieved by the ANN for the calculation of the path considering the *Y* parameter. Table 1 presents a compilation of the comparison between the results achieved by the ANN and by the simple linear extrapolation model. Table 1 also shows the decay in the accuracy of the ANN virtual sensor as variables moved away from their starting point (the failure of the real sensor). The mean X position absolute error of the ANN grew from 0.185 m (at Step 1) to 0.275 m (at Step 8), while the mean *Y* position absolute error of the ANN grew from 0.226 m (at Step 1) to 0.314 m (at Step 8). The *Y* position Nash coefficient remains high for all eight steps, decreasing by just 0.199, while the *X* position Nash coefficient decreased by 0.216. The standard deviations in the *X* and *Y* positions increased by 0.072 and 0.110, respectively, along with the eight steps, which were very similar values to the ones observed in the increase in RMSE in the *X* and *Y* positions, respectively.

Both directions presented average errors, median errors close to zero, and similar standard deviations and RMS. Therefore, there is not enough evidence that the model performed differently along with the two directions. Since both Nash coefficients were above 0.7, this model fared well in tracking for both axes. The path of the database showed more variability for the *Y* axis, as confirmed by the Nash coefficient being more significant for this axis while having a similar mean error.

**Figure 10:**
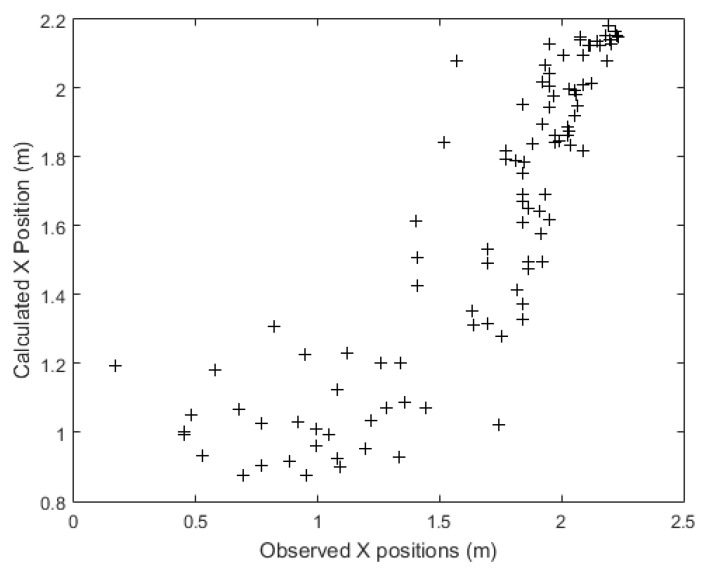
Dispersion of corresponding artificial neural network to the real position considering the *X* axis.

Figure 10 shows the relation between the real position and its corresponding position according to the neural network in respect to the *x* axis. The graph showed less dispersion and better alignment of points with the highest coordinates corresponding to the area closest to the exit from the room, indicating that patient tracking is more accurate the further they were from the door, as illustrated in Figure 7.

**Figure 11:**
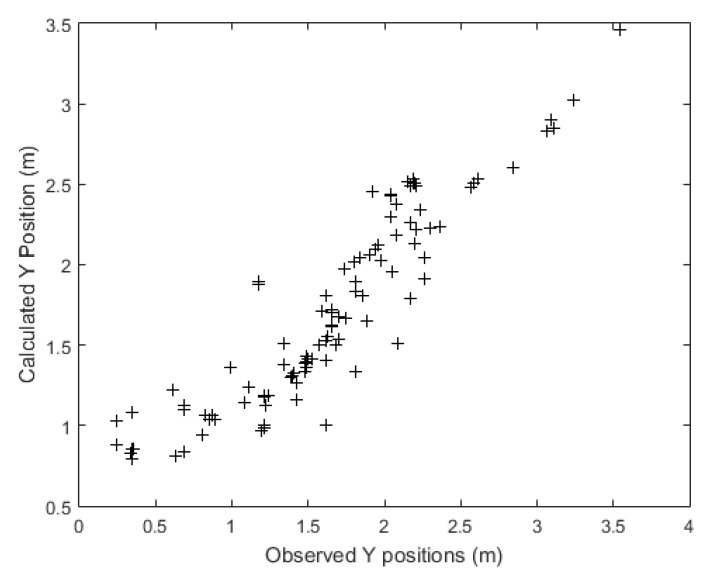
Dispersion of corresponding artificial neural network to the real position considering the *Y* axis.

Figure 11 shows the relation between the real position and its correspondent position according to the neural network with respect to the *y* axis. This graph shows that the diagonal alignment was greater than what was observed for the *x* direction, and the scatter was smaller than what was observed for the *x* coordinates, notably for the lower coordinates.

Since correlation among sensors was already very small (0.305), the virtual sensor could replace the actual sensor for some time, provided that the past position/trajectory was known, and the calculated position is within the active sensor radius. This replacement would work even if the original sensor were already at a greater distance than the one considered between the sensor nodes of the experiments performed in this work, and one of them failed. This correlation was found by comparing the registered data for each of the measured positions from one sensor with the corresponding measured position of the other sensor through MATLAB function *corr*() that establishes the pairwise correlation coefficient between each pair of vectors, which in this work comprised the time series of the positions detected by each sensor.

## 7. Conclusions and Future Work

This paper presented a machine-learning algorithm to replace an indoor-positioning sensor during a faulty period. The number of neurons necessary for the hidden layer of the neural network were also presented. Once appropriately trained, the neural network reproduced the behavior of the sensors well for a few minutes. The used method was reasonably successful, especially for the area close to the exit of a room, which is the most important for security monitoring because it indicates if the patient is leaving a room. Therefore, the usefulness of models that use only one distance sensor and recent patient-position data with artificial neural networks have proven helpful in providing approximate location information when a second sensor occasionally fails.

A possibility of future work considering machine learning is using deep-learning techniques such as transfer learning to further improve the system’s behavior. However, the adoption of this approach may require a greater dataset. In addition, the use of a virtual sensor may also present itself as an interesting solution for remote areas where energy saving is an important concern. Other possibilities could be applying the fault-tolerant approach proposed here in systems exploring spectral diversity (i.e., via broadband measurement [28,29]) or configuration diversity (e.g., via a reconfigurable intelligent surface [28,30]).

## Figures and Tables

**Table 1:** Comparison between ANN and simple linear-extrapolation-model results.

Results	Linear	ANN (t−1)	ANN (t−2)	ANN (t−3)	ANN (t−4)	ANN (t−5)
MAE X (m)	0.263	0.199	0.220	0.219	0.230	0.249
MAE Y (m)	0.572	0.222	0.275	0.299	0.255	0.235
STD X (m)	0.384	0.270	0.303	0.295	0.308	0.323
STD Y (m)	0.752	0.281	0.352	0.384	0.357	0.329
RMSE X (m)	0.382	0.271	0.302	0.296	0.306	0.323
RMSE Y (m)	0.749	0.287	0.351	0.382	0.357	0.331
Nash X	0.438	0.716	0.641	0.657	0.632	0.592
Nash Y	−0.222	0.820	0.727	0.677	0.718	0.758

## Data Availability

Data available on request. The dataset used as input for the performed experiments was obtained from the work reported in [37].

## References

[1] Wyffels J., Goemaere J.P., Verhoeve P., Crombez P., Nauwelaers B., De Strycker L. A novel indoor localization system for healthcare environments. Proceedings of the 2012 25th IEEE International Symposium on Computer-Based Medical Systems (CBMS).

[2] Van Haute T., De Poorter E., Crombez P., Lemic F., Handziski V., Wirström N., Wolisz A., Voigt T., Moerman I. (2016). Performance analysis of multiple Indoor Positioning Systems in a healthcare environment. Int. J. Health Geogr..

[3] Leung C.S., Sum J., So H.C., Constantinides A.G., Chan F.K.W. (2014). Lagrange programming neural networks for time-of-arrival-based source localization. Neural Comput. Appl..

[4] Yan H., Xu Y., Gidlund M. Experimental e-Health Applications in Wireless Sensor Networks. Proceedings of the 2009 WRI International Conference on Communications and Mobile Computing.

[5] Zafari F., Gkelias A., Leung K.K. (2019). A survey of indoor localization systems and technologies. IEEE Commun. Surv. Tutor..

[6] Ouyang R.W., Wong A.K., Lea C., Chiang M. (2012). Indoor Location Estimation with Reduced Calibration Exploiting Unlabeled Data via Hybrid Generative/Discriminative Learning. IEEE Trans. Mob. Comput..

[7] Henningsson M., Tunestål P., Johansson R. (2012). A virtual sensor for predicting diesel engine emissions from cylinder pressure data. IFAC Proc. Vol..

[8] McCulloch W.S., Pitts W. (1943). A logical calculus of the ideas immanent in nervous activity. Bull. Math. Biophys..

[9] Rosenblatt F. (1958). The perceptron: A probabilistic model for information storage and organization in the brain. Psychol. Rev..

[10] Widrow B., Hoff M.E. (1960). Adaptive Switching Circuits.

[11] Rumelhart D.E., Hinton G.E., Williams R.J. (1986). Learning representations by back-propagating errors. Nature.

[12] Hecht-Nielsen R. (1987). Kolmogorov’s mapping neural network existence theorem. Proceedings of the International Conference on Neural Networks.

[13] Hornik K., Stinchcombe M., White H. (1989). Multilayer feedforward networks are universal approximators. Neural Netw..

[14] Hecht-Nielsen R. (1989). Theory of the backpropagation neural network. Neurocomputing.

[15] Alwakeel S.S., Alhalabi B., Aggoune H., Alwakeel M. A machine learning based WSN system for autism activity recognition. Proceedings of the 2015 IEEE 14th International Conference on Machine Learning and Applications (ICMLA).

[16] Mahfouz S., Mourad-Chehade F., Honeine P., Farah J., Snoussi H. (2015). Kernel-based machine learning using radio-fingerprints for localization in wsns. IEEE Trans. Aerosp. Electron. Syst..

[17] Huang H.Y., Hsieh C.Y., Liu K.C., Cheng H.C., Hsu S.J., Chan C.T. (2019). Multi-Sensor Fusion Approach for Improving Map-Based Indoor Pedestrian Localization. Sensors.

[18] Liu M., Chen R., Li D., Chen Y., Guo G., Cao Z., Pan Y. (2017). Scene recognition for indoor localization using a multi-sensor fusion approach. Sensors.

[19] Sarbadhikari S.N., Basak J., Pal S.K., Kundu M.K. (1998). Noisy fingerprints classification with directional FFT based features using MLP. Neural Comput. Appl..

[20] Luo Q., Yan X., Li J., Peng Y., Tang Y., Wang J., Wang D. (2016). DEDF: Lightweight WSN distance estimation using RSSI data distribution-based fingerprinting. Neural Comput. Appl..

[21] Ibrahim A., Rahim S.K.A., Mohamad H. Performance evaluation of RSS-based WSN indoor localization scheme using artificial neural network schemes. Proceedings of the 2015 IEEE 12th Malaysia International Conference on Communications (MICC).

[22] Zhang H., Liu K., Jin F., Feng L., Lee V., Ng J. (2019). A scalable indoor localization algorithm based on distance fitting and fingerprint mapping in Wi-Fi environments. Neural Comput. Appl..

[23] Jiang X., Liu J., Chen Y., Liu D., Gu Y., Chen Z. (2016). Feature Adaptive Online Sequential Extreme Learning Machine for lifelong indoor localization. Neural Comput. Appl..

[24] Vandersmissen B., Knudde N., Jalalvand A., Couckuyt I., Dhaene T., De Neve W. (2019). Indoor human activity recognition using high-dimensional sensors and deep neural networks. Neural Comput. Appl..

[25] Rao X., Li Z. (2019). MSDFL: A robust minimal hardware low-cost device-free WLAN localization system. Neural Comput. Appl..

[26] Qin F., Zuo T., Wang X. (2021). CCpos: WiFi Fingerprint Indoor Positioning System Based on CDAE-CNN. Sensors.

[27] Zhang M., Wang L., Xiong S. (2020). Using Machine Learning Methods to Provision Virtual Sensors in Sensor-Cloud. Sensors.

[28] Del Hougne P., Imani M.F., Fink M., Smith D.R., Lerosey G. (2018). Precise Localization of Multiple Noncooperative Objects in a Disordered Cavity by Wave Front Shaping. Phys. Rev. Lett..

[29] Del Hougne P. (2020). Robust position sensing with wave fingerprints in dynamic complex propagation environments. Phys. Rev. Res..

[30] del Hougne M., Gigan S., del Hougne P. (2021). Deeply Sub-Wavelength Localization with Reverberation-Coded-Aperture. arXiv.

[31] Vasisht D., Kumar S., Katabi D. (2016). Decimeter-Level Localization with a Single WiFi Access Point. Proceedings of the 13th USENIX Symposium on Networked Systems Design and Implementation (NSDI 16).

[32] Kumar S., Gil S., Katabi D., Rus D. (2014). Accurate Indoor Localization with Zero Start-up Cost. Proceedings of the 20th Annual International Conference on Mobile Computing and Networking, MobiCom ’14.

[33] Adib F., Kabelac Z., Katabi D. Multi-Person Localization via RF Body Reflections. Proceedings of the 12th USENIX Conference on Networked Systems Design and Implementation, NSDI’15.

[34] Lampoltshammer T.J., Pignaton de Freitas E., Nowotny T., Plank S., Da Costa J.P.C.L., Larsson T., Heistracher T. (2014). Use of Local Intelligence to Reduce Energy Consumption of Wireless Sensor Nodes in Elderly Health Monitoring Systems. Sensors.

[35] Cardoso D.T., Manfroi D., de Freitas E.P. (2020). Improvement in the Detection of Passengers in Public Transport Systems by Using UHF RFID. Int. J. Wirel. Inf. Netw..

[36] Tavares Bruscato L., Heimfarth T., Pignaton de Freitas E. (2017). Enhancing Time Synchronization Support in Wireless Sensor Networks. Sensors.

[37] Bacciu D., Barsocchi P., Chessa S., Gallicchio C., Micheli A. (2014). An experimental characterization of reservoir computing in ambient assisted living applications. Neural Comput. Appl..

